# Prevalence, Characteristics, and Distribution of Human Papillomavirus According to Age and HIV Status in Women of Eastern Cape Province, South Africa

**DOI:** 10.3390/v16111751

**Published:** 2024-11-08

**Authors:** Zizipho Z. A. Mbulawa, Sinazo Kondlo, Sinalo Toni, Lindiwe M. Faye, Charles B. Businge

**Affiliations:** 1 Department of Laboratory Medicine and Pathology, Walter Sisulu University, Mthatha 5100, South Africa; zizipho.mbulawa@nhls.ac.za (Z.Z.A.M.); 219248966@mywsu.ac.za (S.K.); lfaye@wsu.ac.za (L.M.F.); 2 National Health Laboratory Service, Nelson Mandela Academic Hospital, Mthatha 5100, South Africa; 3 Nelson Mandela Academic Clinical Research Unit (NeMACRU), Mthatha 5100, South Africa; simatoni88@gmail.com; 4 Department of Obstetrics and Gynaecology, Faculty of Health Sciences, Walter Sisulu University, Mthatha 5100, South Africa

**Keywords:** human papillomavirus, human immunodeficiency virus, age, women

## Abstract

Human papillomavirus (HPV) is a sexually transmitted infection associated with the development of cervical cancer. This study investigated cervical HPV prevalence, characteristics, and distribution according to age and human immunodeficiency virus (HIV) status among women attending a public community health facility in the Eastern Cape Province of South Africa. A total of 325 participants (aged 18 to 60) visiting a community health facility for any reason were recruited. Cervical HPV infection was detected using the Seegene Anyplex™ II HPV28 assay (Seegene Inc., Seoul, South Korea). Overall HPV prevalence was 65.2% (95% CI: 59.9–70.2%), with the highest prevalence of 80.9% (95% CI: 67.2–89.8%) observed in the 18–25-year-old age group and the lowest prevalence of 46.3% (95% CI: 35.8–57.1%) in the 46–60-year-old age group. HR-HPV infection was found to decrease with increasing age (*p* < 0.001) in the overall population and according to HIV status. In contrast, LR-HPV infection was found to significantly decrease with age among HIV-negative women (*p* = 0.001) but not for the overall population and HIV-positive women. A proportion of 12.9% were infected with one or more HPV types covered by the Cervarix^®^ HPV vaccine (HPV-16 and/or -18), 18.8% (by those covered by Gardasil^®^4 (HPV-6, -11, -16 and/or -18), and 42.2% by those covered by Gardasil^®^9 (HPV-6, -11, -16, -18, -31, -33, -45, -52 and/or -58). The alpha-9 HPV species was the most dominant species (40.6%), followed by the alpha-7 species (29.8%). High overall HPV, HR-HPV, and alpha-9 species prevalence were observed among the women attending the public health facility. These findings contribute to the limited HPV distribution data among the Eastern Cape women, which could be used to improve HPV-related policy and assess the effectiveness of the HPV vaccination.

## 1. Introduction

Human papillomavirus (HPV) is the most common sexually transmitted viral infection worldwide. Its persistent infection is associated with the development of cancer of the cervix, anus, vagina, vulva, penis, and head and neck [1,2,3]. The majority of the sexually experienced population will acquire an HPV infection at least once in their lifetime [4]. There are more than 200 genetically different HPV genotypes belonging to three genera, namely *Alphapapillomavirus*, *Betapapillomavirus*, and *Gammapapillomavirus* [5,6]. *Alphapapillomavirus* can be divided into high-risk (HR) and low-risk (LR) HPV genotypes; the HR-HPV types are associated with the development of cancer [6,7].

African countries have the highest burden of HPV [8,9,10]. Taku et al. reported an HR-HPV prevalence of approximately 83.0% among Eastern Cape women with high-grade cervical cancer lesions and 26.4% among women (≥30 years) attending community health clinics [11]. Cervical cancer is a major public health problem among women worldwide, with an estimated 660,000 new cases and 350,000 deaths in 2022 [12]. In South Africa, cervical cancer is the second most common cancer [13], and its prevalence remains high despite the free cervical cancer screening services in the health facilities within South Africa [14,15]. The human immunodeficiency virus (HIV) has been associated with a high rate of HPV acquisition, persistent infection, and the development of cervical cancer [16,17,18,19,20]. South Africa has a high burden of HIV [21], which contributes to the high HPV infection, HPV persistent infection, and cervical cancer burden [20]. HIV-positive women are more likely to develop cervical cancer compared to HIV-negative women [16,22].

South Africa introduced a national school-based HPV vaccination program in 2014, targeting girls aged nine years or older and in Grade 4. Since the HPV vaccine introduction, the national school-based HPV vaccination program has followed a two-dose schedule with the Cervarix^®^ HPV vaccine (targets HPV-16 and -18) [13,23,24]. However, HPV vaccine coverage rates in South Africa have been reported to vary and decrease over the years. When the HPV vaccine was introduced in 2014, the coverage for the first dose was 86.0% and 65.0% for the second dose. However, in 2017, the coverage decreased to 69.0% and 56.0%, respectively [23]. In 2021, first dose coverage was 37.0%, while second dose coverage was 34.0%; the coronavirus disease pandemic contributed to the decreased HPV vaccine coverage [13]. Currently, the commercial HPV vaccines are also widely available in private sectors and can be assessed by anyone [23]. The South African cervical cancer screening and control policy indicates that women should receive free cervical cytology screenings starting from 30 years of age using liquid-based cytology at five-year intervals [25]. It further stipulates that high-risk women, such as HIV-positive women, should be screened upon HIV diagnosis and at three-year intervals after that. Although widely available in South African private practice, HR-HPV molecular testing has not yet been fully incorporated as one of the cervical cancer screening methods in the public sector [25,26].

The HPV prevalence and HPV type distributions according to age and HIV status are essential in informing HPV vaccination campaigns and monitoring the impact on HPV types after vaccination. Among the women of the King Sabata Dalindyebo (KSD) municipality, Eastern Cape Province of South Africa, this information is minimal. Therefore, this study investigated cervical HPV prevalence, characteristics, and distribution according to the age and HIV status among women attending a public community health facility in the Eastern Cape Province of South Africa.

## 2. Materials and Methods

### 2.1. Study Setting and Population

This quantitative study was conducted at the health facility in the King Sabata Dalindyelo (KSD) Department of Health sub-district OR Tambo municipality district of the Eastern Cape Province in South Africa. The health facility serves as a primary healthcare facility catering to a diverse population residing in surrounding rural, informal settlements, as well as peri-urban and urban settings. The study population consisted of women who visited the health facility to seek medical attention for any reason, including various conditions, and collect medication. Participants were recruited between June and July 2023 during their visits to the health facility. Participants were recruited at the health facility reception by educating all the health facility attendees using posters and educational leaflets on human papillomavirus and cervical cancer. Recruitment and informed consent processes were conducted in isiXhosa, a locally spoken language. Participation in the study was entirely voluntary, with no obligation or pressure placed upon the individuals to take part. Written consent was obtained from all the study participants. All individuals voluntarily participated in the study, provided biological specimens, and responded to the questionnaires.

### 2.2. Clinical Specimen Collection and HIV Test

Study procedures were conducted in a private room; the investigator attended to all the participants privately. To maintain participant confidentiality, each participant was assigned a unique study number, linking all their collected specimens and questionnaires. An HIV rapid test was offered to all participants who had a negative or unknown HIV status by a qualified nurse or an HIV lay counsellor. Pre- and post-HIV counselling and rapid HIV tests were administered following the South African Department of Health HIV guidelines. The first cervical specimen was collected for cervical cancer screening purposes. It was collected using the ThinPrep Pap Test collection kit (Hologic InC., Marlborough, MA, USA) and sent to the Nelson Mandela Academic Hospital National Health Laboratory Service cytopathology laboratory. The second cervical specimen for HPV testing was then collected using a Digene cervical sampler brush (Qiagen Inc., Gaithersburg, MD, USA) and placed into the Digene transport medium (Qiagen Inc., Gaithersburg, MD, USA). The cervical specimens were transported to the Nelson Mandela Academic Hospital NHLS/WSU virology laboratory and stored at −20 °C until nucleic acid extraction.

### 2.3. Nucleic Acid Extraction and Molecular Detection of HPV

Nucleic acid extraction from the cervical specimens was performed using an automated procedure, Seegene NIMBUS, and a universal STARMag extraction system (Seegene Inc., Seoul, South Korea), following the manufacturer’s instructions. The extracted nucleic acid was used to detect and genotype HPV using Seegene Anyplex™ II HPV28 (Seegene Inc., Seoul, South Korea) multiplexed real-time polymerase chain reaction (PCR). The Seegene Anyplex™ II HPV28 detected, differentiated, and quantified 28 different HPV genotypes (13 HR-HPV: 16, 18, 31, 33, 35, 39, 45, 51, 52, 56, 58, 59, 68; 9 LR-HPV: 6, 11, 40, 42, 43, 44, 54, 61, 70 and 6 Probable HR-HPV: 26, 53, 66, 69, 73, 82). Amplification on a Bio-Rad CFX96 real-time thermocycler (Bio-Rad, Hercules, CA, USA) was conducted according to the manufacturer’s instructions. Anyplex™ II HPV28 targeted the L1 major capsid gene of the HPV types, with the human housekeeping gene (β-globin) as an internal control. The Seegene Viewer Software v3.31.000.006 (Seegene Inc., Seoul, Korea) automatically generated and interpreted the data according to the manufacturer’s instructions.

Samples with a negative internal control and a negative HPV result were re-analyzed; if this persisted, they were deemed invalid. If the internal control was negative, but the HPV test result was positive, the test result was considered valid. Negative HPV tests with a positive internal control were deemed negative for 28 HPV types targeted by the Seegene Anyplex™ II HPV28. A negative control (molecular biology grade water) was included for every 20 samples and taken through the extraction and HPV genotyping step to monitor contamination. In addition to HPV genotype indication, the Seegene Anyplex™ II HPV28 (Seegene Inc. Seoul, South Korea) also provided a semi-quantified HPV viral load. HPV viral load was semi-quantified as high (+++; positive signal before 31 PCR cycles), medium (++; positive signal between 31 and 39 PCR cycles), or low (+; positive signal at and after 40 PCR cycles).

### 2.4. Data AnalysisTable

HPV prevalence was described according to *Alphapapillomavirus* species, HR- and LR-HPV, and type classification. Single HPV infection was defined as infection with one HPV type, while multiple HPV infection was described as being infected with two or more HPV types in the same sample. In addition, HPV types were grouped based on the commercially available HPV vaccines. Women infected with multiple types belonging to the HR-HPV and LR-HPV types were counted more than once, similarly to those infected with types belonging to different *Alphapapillomavirus*. All variables were captured and coded in Microsoft Excel. GraphPad Prism Software v8.0.1.244 statistical software was used to perform all statistical analysis. Numerical data were explored using the Shapiro–Wilk test for normality. The chi-squared test for trends and Fisher’s exact method were used to compare the proportions between variables. The Mann–Whitney U test was used to compare the median between the two groups. The 95% confidence intervals of the proportion were calculated by the modified Wald method (GraphPad Prism). Differences were considered to be statistically significant when *p*-values were <0.05.

## 3. Results

### 3.1. Demographic Characteristics of the Population

A total of 325 women with a median age of 36 years (IQR: 28–45 years; range: 18–60 years) participated in the study. The HIV-positive women (median age 38.5 years, IQR: 32–46 years) were older than the HIV-negative women (median age 31 years, IQR: 23–42 years, *p* < 0.0001, Table 1). Approximately half of the study population had completed secondary education level (53.1%). A high proportion of study participants had a household income of 2000 South African rands or less. All participants were current or once sexually active. The median lifetime sexual partners were four with 3–5 IQR. A majority of the study participants were negative for intraepithelial lesion or malignancy (82.8%), followed by low-grade squamous intraepithelial lesion (LSIL, 4.9%), high-grade squamous intraepithelial lesion (HSIL, 4.3%), atypical squamous cells of undetermined significance (ASCUS, 2.5%), and atypical squamous cells, cannot exclude high grade squamous intraepithelial lesion (ASC-H, 1.2%, Table 1).

### 3.2. HPV Prevalence and Type Distribution According to HIV Status and Age

**Figure 1:**
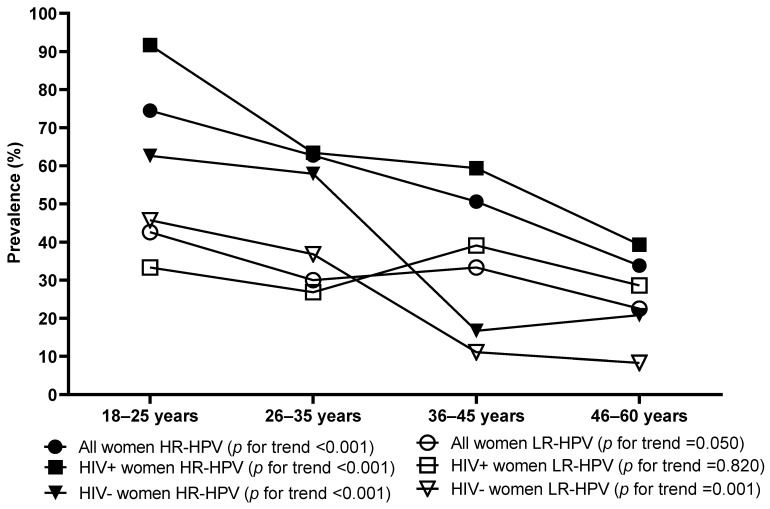
The high-risk and low-risk human papillomavirus (HPV) prevalence by age and HIV status among women of Eastern Cape Province.

Overall HPV prevalence was 65.2% (95% CI: 59.9–70.2%), with the highest prevalence of 80.9% (95% CI: 67.2–89.8%) observed in the 18–25-year-old group and the lowest prevalence of 46.3% (95% CI: 35.8–57.1%) in the 46–60-year-old women. The overall HPV prevalence was not found to significantly differ between the HIV-positive and HIV-negative women (Table 2). Among the HIV-positive women, HPV prevalence was 67.8% (95% CI: 61.2–73.8%), with the highest prevalence of 91.7% (95% CI: 62.5–91.7%) observed in the 18–25-year-old group and the lowest prevalence of 51.8% (95% CI: 39.0–64.3%) in the 46–60-year-old women (Table 2). Among the HIV-negative women, HPV prevalence was 60.0% (95% CI: 50.9–68.5%), with the highest prevalence of 77.1% (95% CI: 60.7–88.2%) observed in the 18–25-year-old women and the lowest prevalence of 27.8% (95% CI: 12.2–51.2%) in the 36–45-year-old women. HPV prevalence was found to decrease with increasing age in all women (*p* for trend <0.0001), HIV-positive (*p* = 0.004), and HIV-negative women (*p* < 0.001). HPV prevalence was high among the HIV-positive women by age group but not statistically different, except for the 36–45-year-old age group (*p* = 0.001, Table 2). The overall HR-HPV prevalence was found to be 53.8% (95% CI: 48.4–59.2%), while LR-HPV was 31.1% (95% CI: 26.3–36.3), and probable HR-HPV was 18.2% (95% CI: 14.3–22.7%). There was no difference in the HR-HPV, probable HR-HPV, and LR-HPV prevalence between the HIV-positive and HIV-negative women (Table 2). HR-HPV infection was found to significantly decrease with increasing age among the overall population (*p* for trend <0.001), HIV-positive women (*p* for trend <0.001), and HIV-negative women (*p* for trend <0.001). In contrast, LR-HPV infection was found to significantly decrease with age among the HIV-negative women (*p* for trend *p* = 0.001) but not for the overall population (*p* for trend =0.050) and the HIV-positive women (*p* for trend =0.820, Figure 1).

**Figure 2:**
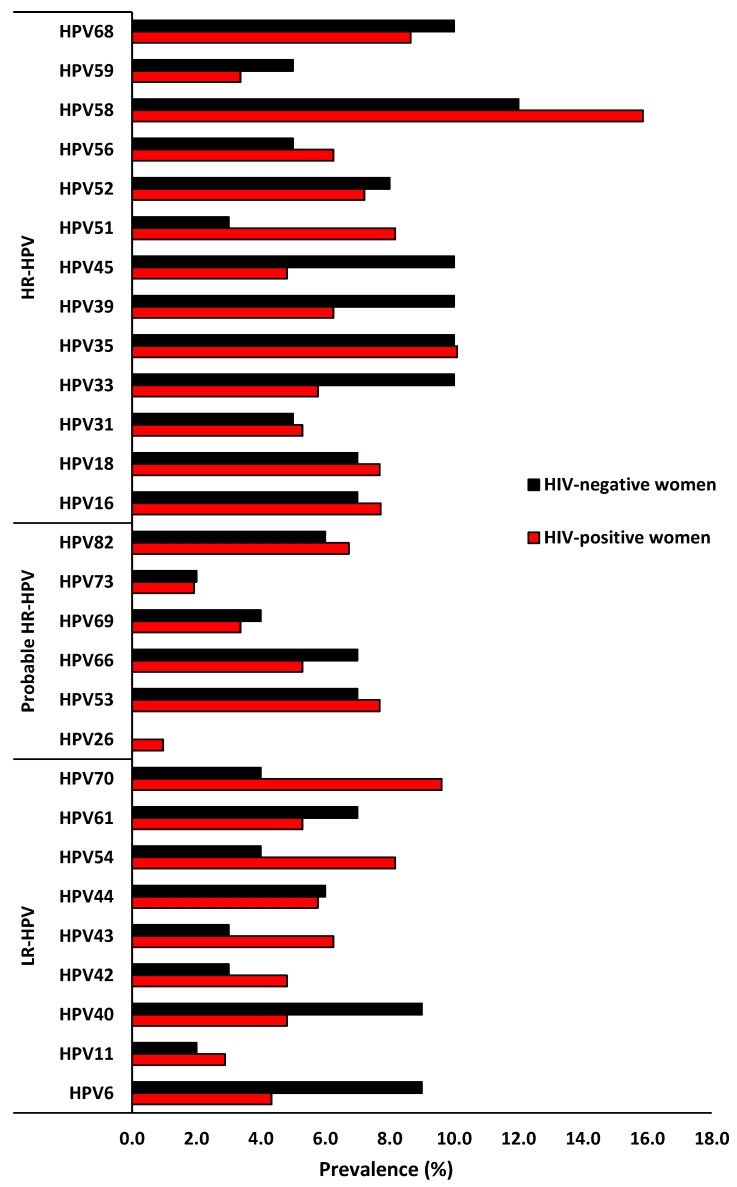
Prevalence and distribution of different human papillomavirus types according to HIV status among women of Eastern Cape Province of South Africa.

Overall, the four most common HR-HPV types were found to be HPV-58 (13.8%), HPV-35 (9.5%), HPV-68 (8.6%), and HPV-16 (7.4%). Among the HIV-positive women, the four most dominant HR-HPV types were HPV-58 (15.9%), HPV-35 (10.1%), HPV-68 (8.7%) and HPV-51 (8.2%). While among the HIV-negative women, the dominant HR-HPV types were HPV-58 (10.4%), HPV-68, -45, -39, -35, -33 (each 8.7%), HPV-52 (7.0%), and HPV-16 and -18 (6.1%, Figure 2). The distribution pattern of the dominant single HR-HPV infection among the HIV-positive women was HPV-58 (3.4%), HPV-68, -16, -52 (1.9% each) and HPV-35, -18, -33 and -31 (1.4%). It is interesting to note that the distribution of the most common HR-HPV types presenting as a single infection was slightly different from the overall infection, and the most dominant single HR-HPV types were HPV-58 (2.6%), HPV-31 (1.7%), HPV-68, -45, -35, and -33 (0.9% each). Among the LR-HPV types, HPV-70 (7.4%), HPV-54 (6.5%), HPV-40 (5.8%), HPV-61, -44, and -6 (5.5% each) were the most dominant types among the overall population (Figure 2).

The distribution remained the same among the HIV-positive women, with HPV-70 (9.6%), HPV-54 (8.2%), HPV-43 (6.3%), and HPV-44 (5.8%), but not among the HIV-negative women, HPV-40, -6 (9.0% each), HPV-61 (7.0%), and HPV-44 (6.0%), HPV-70, and -54 (4.0% each, Figure 2).

### 3.3. The Influence of Age on Single and Multiple HPV Infection Stratified by HIV Status

**Figure 3:**
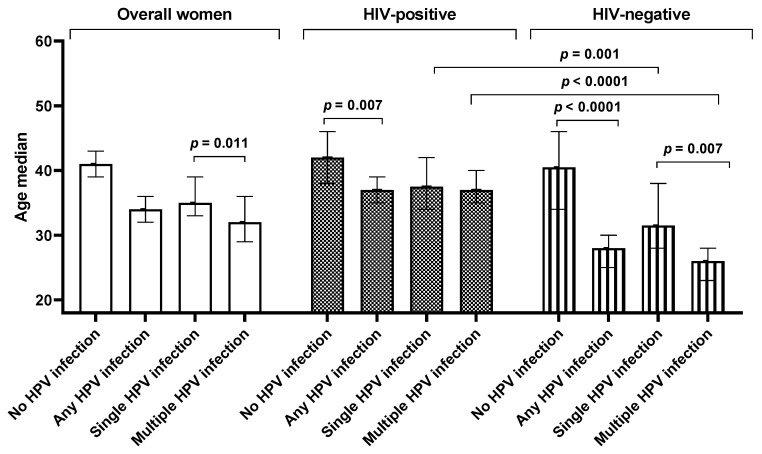
The effect of age and human immunodeficiency virus (HIV) status on overall, single, and multiple human papillomavirus infection among women of Eastern Cape Province.

Overall, women infected with HPV (median: 34 years, IQR: 27–44 years) were significantly younger than those who were HPV negative (median: 41 years, IQR: 32–49 years, *p* < 0.0001). A similar trend was also observed among the HIV-positive women (*p* = 0.007) and HIV-negative women (*p* < 0.0001). Similarly, in the entire sample women with a single HPV infection (median: 35 years, IQR: 31–45 years) were significantly younger than the women without an HPV infection (median: 41 years, IQR: 32–49 years, *p* = 0.013). However, this trend was further observed among HIV-negative women (*p* = 0.025) but not among HIV-positive (*p* = 0.182, Figure 3).

Women with multiple infections (median age: 32 years, IQR: 26–40 years) were younger than women with no infection (median: 41 years, IQR: 32–49 years, *p* < 0.0001). A similar pattern was observed among the HIV-positive women (*p* = 0.002) and HIV-negative women (*p* < 0.0001). The women with a single HPV infection (median: 35 years, IQR: 31–45 years) were older than the women with multiple HPV infections (median: 32 years, IQR: 26–40 years, *p* = 0.011). Among the women with multiple HPV infections, HIV-negative women were younger (median: 26 years, IQR: 22–30 years) than the HIV-positive women (median: 37 years, IQR: 29–42 years, *p* < 0.0001). Similar observations were made among the women with a single HPV infection (median: 32 years, IQR: 28–38 years compared to median: 38 years, IQR: 33–47 years, *p* = 0.001) and overall HPV infection (median: 28 years, IQR: 23–33 years compared to median: 37 years, IQR: 31–44 years, *p* < 0.0001). However, among the HPV-negative women, there was no difference in age between the HIV-positive (median: 42 years, IQR:38–46 years) and HIV-negative women (median: 41 years, IQR: 34–46 years; *p* = 0.601, Figure 3).

### 3.4. Prevalence of HPV Types Targeted by Current Commercial HPV Vaccines

A proportion of 12.9% (42/325) were infected with one or more HPV types covered by the Cervarix^®^ HPV vaccine (HPV-16 and/or -18), 18.8% (61/325) by Gardasil^®^4 HPV types (HPV-6, -11, -16 and/or -18), and 42.2% (137/325) by the Gardasil^®^9 HPV types (HPV-6, -11, -16, -18, -31, -33, -45, -52 and/or -58). The HIV status did not significantly influence the prevalence within these categories (Table 3). The prevalence of infection with types targeted by the Gardasil^®^4 and Gardasil^®^9 HPV vaccine types were found to decrease with increasing age (*p* = 0.009 and *p* = 0.001) but not with the Cervarix^®^ HPV vaccine types (*p* = 0.074, Table 3).

### 3.5. Alphapapillomavirus Species Prevalence According to HIV and Cervical Cytology

**Figure 4:**
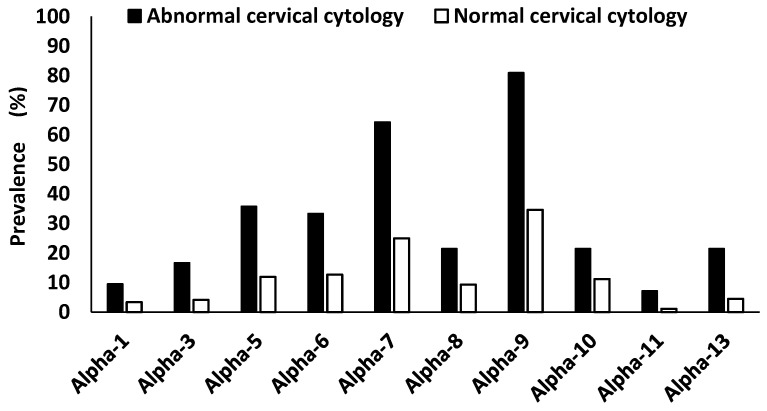
The prevalence of *Alphapapillomavirus* species according to cervical cytology among women of Eastern Cape Province. Alpha-1: HPV-42. Alpha-3: HPV-61. Alpha-5: HPV-26, -51, -69, -82. Alpha-6: HPV-53, -56, -66. Alpha-7: HPV-18, -39, -45, -59, -68, -70. Alpha-8: HPV-40, -43. Alpha-9: HPV-16, -31, -33, -35, -52, -58. Alpha-10: HPV-6, -11, -44. Alpha-11: HPV-73. Alpha-13: HPV-54.

Overall, the alpha-9 HPV species was the most dominant (40.6%, 132/325), followed by the alpha-7 species (29.8%, 97/325), alpha-6 (15.4%, 50/325), alpha-5 (14.8%, 48/325), alpha-10 species (12.3%, 40/325), alpha-13 (6.5%, 21/325), alpha-3 (5.5%, 18/325), alpha-1 (4.0%, 13/325), and alpha-11 (1.8%, 6/325). The alpha-9 and alpha-7 HPV species remain dominant among HIV-positive women (43.3%, 90/208; 34.6%, 72/208, respectively), and among the HIV-negative women (35.7%, 41/115; 20.9%, 24/115, respectively). Up to 60.6% (163/269) of the women who were negative for intraepithelial lesion or malignancy (NILM) were found to be HPV positive, and 47.2% (127/269) were found to be positive with HR-HPV types. All the women who had abnormal cervical cytology (ASCUS, LSIL, ASC-H and HSIL) were HPV infected (100.0%, 42/42), and 97.6% (41/42) were infected with HR-HPV types. Women with abnormal cervical cytology were more likely to have higher semi-quantified HPV viral load than the women who were negative for intraepithelial lesion or malignancy (*p* < 0.0001). Due to the small sample size, this was not further stratified according to HIV status and age. Among the women with abnormal cervical cytology, the HPV alpha-9 species were the most dominant species (81.0%, 34/42), followed by the alpha-7 species (64.3%, 27/42), alpha-5 species (35.7%, 15/42), alpha-6 species (33.3%, 14/42), alpha-8 species, and alpha-13 species (21.4%, 9/42 each), alpha-3 species (16.7%, 7/42), alpha-1 species (9.5%, 4/42), and alpha-11 species (7.1%, 3/42, (Figure 4).

## 4. Discussion

According to our knowledge, this is the first report on cervical HR, LR, probable HR-HPV, and *Alphapapillomavirus* species prevalence and distribution according to age and HIV status among women attending a public community health facility in the Eastern Cape Province of South Africa. There was a high overall HPV and HR-HPV prevalence even though 82.8% of the participants had normal cervical cytology. An HIV-positive status is commonly associated with increased HPV prevalence [19,27]; however, in the current study, there was no difference between the HIV-negative and HIV-positive women. This could be due to the high proportion of HIV-negative women who were significantly younger than the HIV-positive women. The literature has reported that HPV prevalence tended to be higher among the younger age groups [28,29]; similar findings were observed in the current study. In addition, in the current study, the HPV prevalence was similar between the HIV-positive and HIV-negative women at 18–25 years and 26–35 years of age, whereas it was higher among the HIV-positive women at 36–45 years and 46–60 years of age than the HIV-negative women. The current study observed that the younger individuals had different distributions of HPV types than the older individuals. This could be due to different factors like sexual activity patterns, immune system changes, or exposure to other HPV types over time [30].

It was interesting to note that HPV-58 was the most dominant type for both the multiple infection and single infection formats among the overall study population, HIV-positive women, and HIV-negative women. In Southern Africa, HPV-58 is not commonly reported as the most dominant HPV type among women with normal cervical cytology [10,31]. We previously reported HPV-58 as the most dominant type among the Western Cape Province’s adolescents [32]. However, dominant HPV-58 prevalence has been reported among black women residing in the rural community of Brazil [33] and among Chinese women with normal cervical cytology [34]. HPV-35 was the second most dominant type in the overall population and among HIV-positive women. HPV-35 is among the HR-HPV types not covered in the current commercial HPV vaccines (Cervarix^®^, Gardasil^®^4 and Gardasil^®^9) despite being highly prevalent in populations with African ancestry origin with HSIL [35,36,37,38]. Okeke (2024) explained the urgent need for HPV-35 genomic epidemiology among women of African ancestry and the justification of the necessity of a commercial HPV vaccine targeting HPV-35 [36]. Almost half of the study population were infected with HPV types targeted by the Gardasil^®^9 HPV vaccine, showing that South African women will greatly benefit from the currently commercially available HPV vaccines targeting a high number of HPV types.

The alpha-9 and -7 species were the most dominant species among the overall population, HIV-positive women, HIV-negative women, and women with abnormal and normal cervical cytology. HPV types belonging to alpha-9 and -7 were mostly HR-HPV types associated with the majority of cervical cancer cases, and seven of these types (HPV-16, -18, -31, -33, -45, -52 and -58) are targeted by the Gardasil^®^9 HPV vaccine [39]. Zhao et al. (2022) reported that women infected with the alpha-9 HPV species had an increased incidence of HSIL compared to when they were infected with other alpha HPV species [40]. All women who had abnormal cervical cytology were infected with HPV, and almost all of them had HR-HPV types. More than 80% of these women were infected with alpha-9 HPV types, which demonstrates a strong association between HR-HPV and alpha-9 HPV type infection and the high predisposition to the development of abnormal cervical cells, which are precursors to cervical cancer [1,3].

The cervical cancer screening uptake by South African women has also been reported to be low [14,41]. In the South African province where the study was conducted, the knowledge about HPV and its associated cervical cancer is very limited [41,42]. It is of high concern that the current study observed very high overall HPV, HR-HPV, and alpha-9 species prevalence among the Eastern Cape Province women regardless of HIV status. These findings underscore the importance of regular cervical cancer screening not only among HIV-positive women but for all women.

Several factors could act as the limitations of the current study. First, this study is a cross-sectional study, and a longitudinal study could provide a very detailed picture of the reported HPV profile among the Eastern Cape women. Secondly, the small sample size could be another limitation. Thirdly, the study was performed in only one health facility. Enrolment of participants from several health centers spread all over the Eastern Cape Province would yield a sample more representative of the target population. Despite these limitations, the findings of the study remain interesting and important for the Eastern Cape Province and South Africa.

## 5. Conclusions

High overall HPV, HR-HPV, and alpha-9 species prevalence despite the HIV status were observed among women attending the public health facility. The high prevalence of HR-HPV types among women without cervical lesions underscores the urgent need to implement cervical cancer screening using an HPV-DNA/mRNA test. These findings contribute to the limited HPV distribution data among the Eastern Cape women. The South African Department of Health’s policymakers could use the study’s findings to improve HPV-related policies and assess the effectiveness of the HPV vaccination.

## Figures and Tables

**Table 1:** Study participants’ characteristics.

Characteristic	%	n/N
**Age groups**		
18–25 years	14.5	47/325
26–35 years	33.8	110/325
36–45 years	26.8	87/325
46–60 years	24.6	80/325
Missing	0.3	1/325
**HIV status**		
HIV-positive	64.0	208/325
HIV-negative	35.4	115/325
Missing	0.6	2/325
**Lifetime sexual partners, Median (IQR)**	4 (3–5)	
**Education level**		
Primary	6.5	21/324
Secondary	53.1	172/324
Tertial	40.4	131/324
**Income, South African Rands**		
≤R2 000	62.2	201/323
R2 001–R4 000	18.9	61/323
R4 001–R9 000	14.2	46/323
R10 000–R25 000	4.6	15/323
**Cervical cytology**		
NILM ^1^	82.8	269/325
ASCUS ^2^	2.5	8/325
LSIL ^3^	4.9	16/325
ASC-H ^4^	1.2	4/325
HSIL ^5^	4.3	14/325
CDD ^6^	4.0	13/325
Missing	0.3	1/325

^1^ Negative for intraepithelial lesion or malignancy, ^2^ Atypical squamous cells of undetermined significance, ^3^ Low grade squamous intraepithelial lesion, ^4^ Atypical squamous cells, cannot exclude high-grade squamous intraepithelial lesion, ^5^ High-grade squamous intraepithelial lesion, ^6^ Cytology diagnosis deferred.

**Table 2:** Prevalence of single and multiple human papillomavirus (HPV) infections according to HIV status and age among women of Eastern Cape Province of South Africa.

	All Women			HIV-Positive Women			HIV-Negative Women			
	%	95% CI	n/N	%	95% CI	n/N	%	95% CI	n/N	*p*-Value *
**Overall HPV**	65.2	59.9–70.2	212/325	67.8	61.2–73.8	141/208	60.0	50.9–68.5	69/115	0.181
**Overall HPV by age groups**										
18–25 years	80.9	67.2–89.8	38/47	91.7	62.5–99.9	11/12	77.1	60.7–88.2	27/35	0.412
26–35 years	73.6	64.7–81.0	81/110	71.8	60.4–81.0	51/71	76.3	60.6–87.2	29/38	0.656
36–45 years	63.2	52.7–72.6	55/87	72.5	60.9–81.7	50/69	27.8	12.2–51.2	5/18	**0.001**
46–60 years	46.3	35.8–57.1	37/80	51.8	39.0–64.3	29/56	33.3	17.8–53.4	8/24	0.149
*p* for trend		***p* < 0.0001**			***p* = 0.004**			***p* < 0.001**		
**Single HPV infection**	26.5	22.0–31.5	86/325	26.9	21.3–33.3	56/208	24.3	17.4–33.0	28/115	0.692
**Dual HPV infection**	14.2	10.8–18.4	46/325	16.8	12.3–22.5	35/208	9.6	5.3–16.5	11/115	0.096
**Triple HPV infection**	10.5	7.6–14.3	34/325	10.1	6.6–15.0	21/208	11.3	6.6–18.5	13/115	0.710
**Quadruple+ HPV infection ^#^**	14.2	10.8–18.4	46/325	13.9	9.8–19.4	29/208	14.8	9.3–22.5	17/115	0.869
**Multiple HPV infection**	38.8	33.6–44.2	126/325	40.9	34.4–47.7	85/208	35.7	27.5–44.8	41/115	0.405
**HR-HPV**	53.8	48.4–59.2	175/325	56.7	49.9–63.3	118/208	47.8	38.9–56.9	55/115	0.131
**Probable HR-HPV**	18.2	14.3–22.7	59/325	17.8	13.2–23.6	37/208	19.1	12.9–27.3	22/115	0.766
**LR-HPV**	31.1	26.3–36.3	101/325	32.2	26.2–38.8	67/208	29.6	22–38.5	34/115	0.707

* compares HIV-positive and HIV-negative women. ^#^ refer to four and above HPV types.

**Table 3:** Prevalence of human papillomavirus (HPV) types targeted by current commercial HPV vaccines according to HIV status and age.

	Cervarix^®^		Gardasil^®^4		Gardasil^®^9	
	n	%	n	%	n	%
All women, n = 325	42	12.9	56	17.2	137	42.2
HIV-positive women, n = 208	28	13.5	37	17.8	92	44.2
HIV-negative women, n = 115	13	11.3	18	15.7	45	39.1
18–25 years, n = 47	8	17.0	14	29.8	28	59.6
26–35 years, n = 110	16	14.5	19	21.8	51	46.4
36–45 years, n = 87	13	14.9	14	16.1	34	39.1
46–60 years, n = 80	5	6.3	8	10.0	24	30.0

Cervarix^®^ HPV vaccine targets HPV–16/18; Gardasil^®^4 HPV vaccine targets HPV–6/11/16/18; and Gardasil^®^9 HPV vaccine targets HPV–6/11/16/18/31/33/45/52/58).

## Data Availability

Data is attached as Supplementary to this article.

## Supplementary Materials

The following supporting information can be downloaded at: https://www.mdpi.com/article/10.3390/v16111751/s1.
